# Impairment of Tomato *WAT1* Enhances Resistance to Vascular Wilt Fungi Despite Severe Growth Defects

**DOI:** 10.3389/fpls.2021.721674

**Published:** 2021-09-13

**Authors:** Katharina Hanika, Danny Schipper, Shravya Chinnappa, Marian Oortwijn, Henk J. Schouten, Bart P. H. J. Thomma, Yuling Bai

**Affiliations:** ^1^Plant Breeding, Wageningen University & Research, Wageningen, Netherlands; ^2^Laboratory of Phytopathology, Wageningen University & Research, Wageningen, Netherlands

**Keywords:** *Verticillium*, *Fusarium*, susceptibility gene, resistance breeding, pleiotropic effect

## Abstract

*Verticillium dahliae* is a particularly notorious vascular wilt pathogen of tomato and poses a reoccurring challenge to crop protection as limited qualitative resistance is available. Therefore, alternative approaches for crop protection are pursued. One such strategy is the impairment of disease susceptibility (*S*) genes, which are plant genes targeted by pathogens to promote disease development. In Arabidopsis and cotton, the *Walls Are Thin 1* (*WAT1*) gene has shown to be a *S* gene for *V. dahliae*. In this study, we identified the tomato *WAT1* homolog Solyc04g080940 (*SlWAT1*). Transient and stable silencing of *SlWAT1*, based on virus-induced gene silencing (VIGS) and RNAi, respectively, did not consistently lead to reduced *V. dahliae* susceptibility in tomato. However, CRISPR-Cas9 tomato mutant lines carrying targeted deletions in *SlWAT1* showed significantly enhanced resistance to *V. dahliae*, and furthermore also to *Verticillium albo-atrum* and *Fusarium oxysporum* f. sp. *lycopersici* (*Fol*). Thus, disabling the tomato *WAT1* gene resulted in broad-spectrum resistance to various vascular pathogens in tomato. Unfortunately these tomato CRISPR mutant lines suffered from severe growth defects. In order to overcome the pleiotropic effect caused by the impairment of the tomato *WAT1* gene, future efforts should be devoted to identifying tomato *SlWAT1* mutant alleles that do not negatively impact tomato growth and development.

## Introduction

Vascular wilt pathogens cause diseases in many annual and perennial crops (Yadeta and Thomma, 2013). Vascular pathogens of tomato include fungi such as *Fusarium* (Michielse and Rep, 2009) and *Verticillium* (Fradin and Thomma, 2006), as well as bacteria such as *Clavibacter* (Nandi et al., 2018), *Ralstonia* (Peeters et al., 2013), and *Xanthomonas* (Potnis et al., 2015). Vascular pathogens are hard to combat once they invaded a plant host (Yadeta and Thomma, 2013). The soil-borne fungus *Verticillium dahliae* is particularly hard to control due to its wild host range that comprises hundreds of hosts and its persisting resting structures in the soil (Fradin and Thomma, 2006). Crop protection therefore relies on the use of resistant plant varieties. For *V. dahliae* only one monogenic resistance gene, the *Ve1* tomato gene, has been cloned so far (Fradin et al., 2009). This resistance is based on the recognition of the *V. dahliae* avirulence protein Ave1 by the resistance (R) protein encoded by the *Ve1* gene (de Jonge et al., 2012). However, this resistance has been overcome by *V. dahliae* strains that have purged the *Ave1* gene, posing a reoccurring challenge for tomato cultivation (Grogan, 1979; Dobinson et al., 1996; de Jonge et al., 2012; Usami et al., 2017).

To address the recurrent problem of the breakdown of *R* gene-mediated resistance, alternative approaches can be pursued, such as the impairment of disease susceptibility (*S*) genes (Pavan et al., 2010; Gawehns et al., 2013). *S* genes are host genes that play an important role in disease establishment by the pathogen. *S* genes can function in a multitude of ways, including early recognition of the pathogen, negative regulation of immune responses, or pathogen sustenance (van Schie and Takken, 2014). Nevertheless, *S* genes also have functions for the host. *S* gene-mediated resistance, or rather loss-of-susceptibility, is achieved by circumventing the manipulation of these gene products by the pathogen, preferably whilst keeping the intrinsic function for the host intact. In wild germplasm such impaired *S* gene alleles can occur naturally, for example as loss-of-function mutations or as promoter mutations leading to impaired expression (Chu et al., 2006; Bai et al., 2008; Gao et al., 2015). Alternatively, these impairments can be introduced by random mutagenesis or by targeted genome editing, for example using CRISPR-Cas9 (Zaidi et al., 2018; Dong and Ronald, 2019). Impairment of *S* genes can be associated with severe pleiotropic effect as a consequence of not only impairment of its function for the pathogen, but also impairment of its intrinsic role for the host. For instance for the *defense no death 1* (*dnd1*) mutant, loss-of-susceptibility to *Pseudomonas syringae* is accompanied by dwarfism in Arabidopsis and tomato, and spontaneous lesion formation in potato (Sun et al., 2016). An important benefit, however, is that impairment of *S* genes can lead to non-race specific resistance to different strains of a given pathogen (Jørgensen, 1992), or even to broad-spectrum resistance to multiple pathogens (Wang et al., 2018). This illustrates the potential of *S-*gene mediated resistance in crop protection.

An example of broad-spectrum resistance to different vascular pathogens is provided by the *walls are thin 1* (*wat1*) mutant (Denancé et al., 2013). This mutant was identified in an Arabidopsis screen for cell wall mutants (Ranocha et al., 2010), and displays resistance to the bacteria *Ralstonia solanacearum* and *Xanthomonas campestris*, and the fungi *V. dahliae, Verticillium albo-atrum*, and *Plectoshaerella cucumerina* (Denancé et al., 2013). *WAT1* encodes a tonoplast-localized auxin transporter (Ranocha et al., 2013), but its exact role in so-called “vascular immunity” is not yet understood. *WAT1* orthologs have been characterized in several plant species (Ranocha et al., 2010), and recently its role as susceptibility factor in cotton was investigated, demonstrating that simultaneous transient silencing of three *WAT1* homologs resulted in increased resistance to *V. dahliae* (Tang et al., 2019). In this study, we aimed to identify the tomato *WAT1* ortholog and examine its role as a susceptibility factor in tomato for vascular pathogens such as *Verticillium* spp. and *Fusarium oxysporum.*

## Materials and Methods

### Plant and Fungi Growth Conditions

All tomato genotypes were grown in the greenhouse (Unifarm, Wageningen University & Research, Netherlands) at 21/19°C (day/night) at 60% relative humidity and a minimal light intensity of 100 W/m^2^ in potting soil (Potgrond 4, Horticoop, Katwijk, Netherlands). *V. dahliae* (strain JR2, race 1), *V. albo-atrum* (strain CBS385.91, race 1) and *F. oxysporum* f. sp. *lycopersici* (stain Bt.01, race 1) were maintained on potato dextrose agar (PDA) at room temperature in the dark.

### Virus-Induced Gene Silencing

Virus-induced gene silencing (VIGS) was carried out as described previously using tobacco rattle virus (TRV) (Liu et al., 2002; Fradin et al., 2009; Verlaan et al., 2013). Briefly, a gene-specific 150–300 bp fragment was amplified using Phusion High-Fidelity DNA polymerase (New England Biolabs, Bioké, Leiden, Netherlands) with primers mentioned in Supplementary Table 1. The obtained fragment was cloned into the tobacco rattle virus 2 (TRV2) vector (Liu et al., 2002) using Gateway cloning and subsequently transformed into *Agrobacterium tumefaciens* strain GV3103. As negative control a TRV2 vector containing a fragment of the *β-Glucuronidase* (*GUS*) gene was used (Wu et al., 2011; Senthil-Kumar and Mysore, 2014). Moreover, a TRV2 vector carrying a fragment of the tomato *phytoene desaturase* (*PDS*) gene was used as a positive control as it triggers photobleaching upon effective silencing.

### Generation of CRISPR-Cas9 and RNAi Lines

To design single guide RNAs (sgRNAs) the ‘‘CCTop -- CRISPR/Cas9 target online predictor’’^1^ (Stemmer et al., 2015) was used and for target site evaluation the tomato genome (*Solanum lycopersicum* Solyc2.5) was used as reference. Only sgRNAs with a maximum of one exonic off-target site were selected. All sgRNAs were verified to contain a GC-content^2^ between 30 and 80% and presence of required secondary structures was evaluated^3^ (Zuker, 2003) according to Liang et al., 2016. Different scoring tools^4^,^5^,^6^ (Wong et al., 2015; Chari et al., 2017; Sanson et al., 2018) were used to select the best sgRNAs which met most of the criteria. In total, four sgRNAs were designed (Supplementary Table 1).

Golden Gate Cloning (Engler et al., 2008) was used to clone the constructs, and plasmids were obtained from Addgene^7^ : pICH86966 (level 0 plasmid for amplification), pICSL01009 (level 0 plasmid containing AtU6), pICH47751 (level 1 sgRNA1), pICH47761 (level 1 sgRNA2), pICH47772 (level 1 sgRNA3), pICH47781 (level 1 sgRNA4), pICH47732 (level 1 containing NPTII), pICH47742 (level 1 containing Cas9), pICH41822 (linker), and pAGM4723 (level 2 binary vector) (Weber et al., 2011). Phusion High-Fidelity DNA Polymerase (Thermo Scientific, Bleiswijk, Netherlands) was used to amplify sgRNAs, and PCR products were purified with QIAquick PCR Purification Kit (Qiagen Benelux B.V., Venlo, Netherlands). Level 1 plasmids were digested using *Bsa*I/*Eco*31I and ligated using T4 DNA ligase (Thermo Scientific, Bleiswijk, Netherlands) and cloned into *Escherichia coli* strain DH5α (Thermo Scientific, Bleiswijk, Netherlands). Plasmids were purified using QIAprep Spin Miniprep Kit (Qiagen Benelux B.V., Venlo, Netherlands). Level 2 plasmids were digested using *Bpi*I/*Bps*I and ligated using T4 DNA ligase (Thermo Scientific, Bleiswijk, Netherlands), cloned into *E. coli* strain DH5α, purified and sequenced. All plasmids were cloned into *A. tumefaciens* strain AGL1+virG. Transformation of tomato cultivar Moneymaker (MM) was carried out as described previously (Huibers et al., 2013).

To generate the *WAT1* RNAi construct, the same fragment that was used for VIGS was cloned from the TRV2 vector into the pHellsgate8 vector (Helliwell and Waterhouse, 2003) using Gateway cloning (Katzen, 2007). Subsequently the construct was transformed into *A. tumefaciens* strain AGL1+virG. Tomato transformation of cultivar MM was carried out as described previously (Huibers et al., 2013).

### Pathogen Inoculations, Phenotyping, and Fungal Biomass Quantification

*Verticillium dahliae, V. albo-atrum*, and *F. oxysporum* (*Fol*) inoculations were carried out with root dipping as described previously (Fradin et al., 2009; Boshoven, 2017). For phenotyping, stunting (%) between inoculated and mock-inoculated plants was calculated based on plant canopy area at 21 days post inoculation (dpi) using ImageJ (Abramoff et al., 2004) as follows:


$$stunting\left(\%\right)=\left(1-\frac{c\,a\,n\,o\,p\,y\,a\,r\,e\,a\,o\,f\,V.d\,a\,h\,l\,i\,a\,e\,i\,n\,o\,c\,u\,l\,a\,t\,e\,d\,p\,l\,a\,n\,t\,s}{a\,v\,e\,r\,a\,g\,e\,c\,a\,n\,o\,p\,y\,a\,r\,e\,a\,o\,f\,m\,o\,c\,k\,t\,r\,e\,a\,t\,e\,d\,p\,l\,a\,n\,t\,s}\right)\times 100\%$$

To quantify fungal biomass, stems sections (∼ 2 cm around the cotyledons) were harvested at 21 dpi and freeze-dried for 48 h. Subsequently, material was ground, and DNA was isolated using CTAB buffer (200 mM Tris–HCl pH 7.5, 50 mM EDTA pH 8.0, 2 M NaCl, 2% CTAB). Fungal biomass was determined on genomic DNA targeting the *ITS* gene (*V. dahliae, V. albo-atrum*, and *F. oxysporum*) relative to the reference gene *SlRUB* (Supplementary Table 1) with the CFX96 Real-time System (Bio-Rad, Veenendaal, Netherlands) and SYBR Green Master Mix (Bio-Rad, Veenendaal, Netherlands). Data were normalized to MM with the 2^–ΔΔCt^ method (Livak and Schmittgen, 2001).

### DNA Isolation and Genotyping

To genotype RNAi and CRISPR plants, DNA was isolated from young leaves using CTAB buffer (1 M Tris–HCl pH 7.5, 0.5 M EDTA pH 8.0, 5 M NaCl, 2% CTAB). PCR was performed with DreamTaq DNA polymerase (Thermo Scientific, Bleiswijk, Netherlands) and corresponding primers (Supplementary Table 1). PCR products were sequenced by Marcrogen Europe (Amsterdam, Netherlands).

### RNA Isolation and Quantitative Reverse Transcription PCR

To quantify silencing levels in RNAi plants, root material was harvested at 21 dpi and snap-frozen in liquid nitrogen. Total RNA was isolated with the MagMAX-96 Total RNA Isolation Kit (Invitrogen, Bleiswijk, Netherlands) using a KingFisher Flex System (Thermo Scientific, Bleiswijk, Netherlands) and synthesis of cDNA was performed with the iScript cDNA Synthesis Kit (Bio-Rad, Veenendaal, Netherlands) according to the manufacturer’s instructions. Quantitative reverse transcription PCR (qRT-PCR) was carried out with the CFX96 Real-time System (Bio-Rad, Veenendaal, Netherlands) and SYBR Green Master Mix (Bio-Rad, Veenendaal, Netherlands) according to the manufacturer’s instructions. Gene expression was determined using the 2^–ΔΔCt^ method (Livak and Schmittgen, 2001) relative to the *tomato elongation factor 1 α* (*SlEF1α*) (Supplementary Table 2). Data were normalized to transformants devoid of the silencing construct or, when not available, to MM plants.

## Results

### Transient Silencing of *WAT1* in Tomato Might Reduce Susceptibility to *V. dahliae*

**FIGURE 1:**
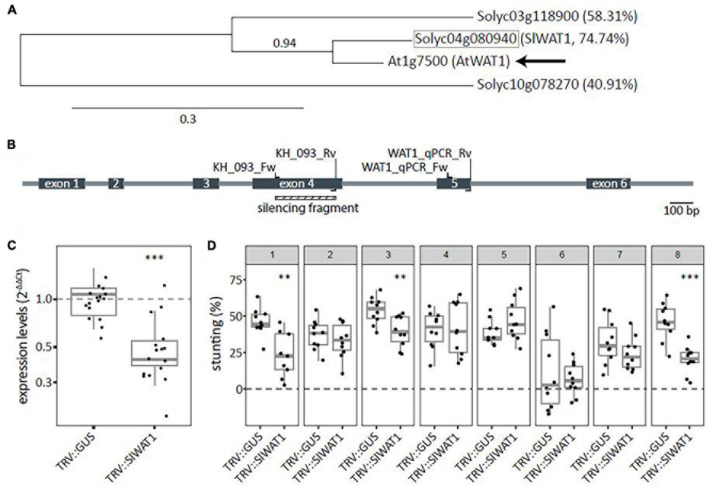
Transient silencing of *WAT1* in tomato resulted in reduced susceptibility to *Verticillium dahliae*. **(A)** Phylogenetic tree based on amino acid sequences for *WAT1* including potential ortholog from tomato (highlighted in gray square). Percentages indicate protein sequence similarity to AtWAT1 marked with an arrow. Number above node indicate branch support values. **(B)** Schematic overview of *SlWAT1* (Solyc04g080940) with intron/exon locations as well as primers for cloning the VIGS fragment and for monitoring the *SlWAT1* expression. **(C)** Expression levels (2^–ΔΔCt^) of plants treated with TRV::SlWAT1 in stems normalized to TRV::GUS 2 weeks after *Agrobacterium tumefaciens* treatment on a log10 scale. Data of two independent experiments with *n* ≥ 6 per experiment per genotype (*t*-test when compared with TRV::GUS with ^∗∗∗^*p* < 0.001). **(D)** Stunting (%) in *Verticillium dahliae*-inoculated plants when compared with the average of mock-inoculated plants at 21 dpi after transient silencing of *SlWAT* in eight independent experiments with *n* ≥ 8 per experiment per genotype (*n* ≥ 8, *t*-test when compared with TRV::GUS with ^∗∗^*p* < 0.01 and ^∗∗∗^*p* < 0.001).

To identify tomato *WAT1* orthologs, the amino acid sequence of the Arabidopsis *WAT1* gene (At1g75500) was obtained from TAIR^8^ and used as query in a blastP search against the Sol genomics database (ITAG release 4.0^9^). Two close homologs were identified and phylogenetic trees were constructed using Phylogeny.fr (Dereeper et al., 2008). Results showed that the tomato gene Solyc04g080940 (hereafter *SlWAT1*) has the highest homology of 74.7% to *AtWAT1* (Figure 1A). To functionally test the *SlWAT1* gene for a role as *S* gene in tomato, *A. tumefaciens*-mediated VIGS was used for transient silencing of *SlWAT1.* One TRV construct (TRV::SlWAT1) was made (Figure 1B), which resulted in a significant reduction in relative expression of *SlWAT1* to approximately 49.6% in plants treated with TRV::SlWAT1 when compared with TRV::GUS-treated plants (Figure 1C). To screen for reduced susceptibility to *V. dahliae* resulted from silencing *SlWAT1*, stunting based on canopy area was calculated between mock- and *V. dahliae*-inoculated plants at 21 dpi. Compared with *V. dahliae-*inoculated TRV::GUS plants, *V. dahliae-*inoculated plants treated with the TRV::SlWAT1 construct showed significantly less stunting in three out of the eight performed experiments (Figure 1D, panels 1, 3, and 8).

### RNAi Knock-Down of *SlWAT1* Did Not Confirm Loss of Susceptibility to *V. dahliae*

Since the silencing effect *via* VIGS was transient and patchy, an RNAi approach was taken to further verify the role of *SlWAT1* in *V. dahliae* susceptibility. An RNAi construct was made with the VIGS fragment (Figure 1A) and used to transform the tomato cultivar MM. Several primary transformants (T1) were evaluated by testing for stable integration of the silencing construct and by determining residual *WAT1* expression levels. In the T1 transformants relative *WAT1* expression varied greatly, from 11 to 270%, when compared with the expression levels found in leaves of control plants (Supplementary Table 2). Five T1 transformants with reduced *SlWAT1* expression were transferred to the greenhouse for T2 seed production. However, seeds were only obtained from three transformants, TV181034, TV181036, and TV181037, which were used for further study (Supplementary Table 2).

**FIGURE 2:**
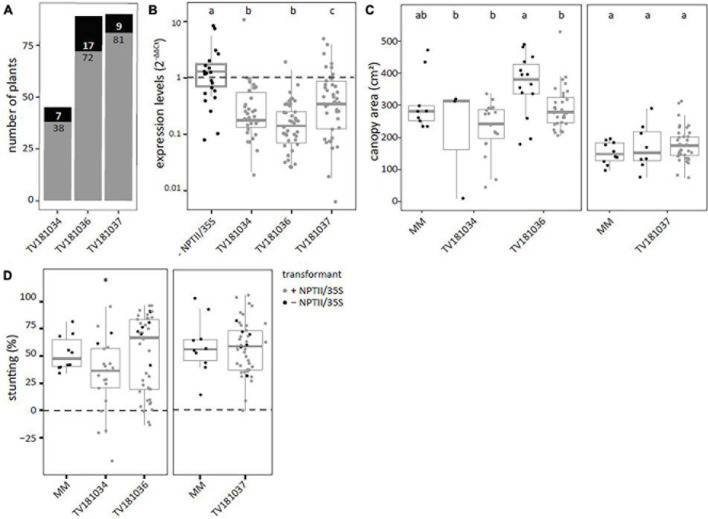
Reduced susceptibility to *V. dahliae* was found in *SlWAT1* T2 RNAi family TV181034, but not for TV181036 and TV181037. **(A)** Total number of plants with (gray) and without (black) the silencing construct for each of the obtained T2 RNAi families. **(B)** Expression levels of *SlWAT1* in roots collected at 21 dpi of plants with the silencing construct (+ NPTII/35S, gray dots) relatively to plants without the silencing construct (– NPTII/35S, black dots) for all families. Data were normalized using 2^–ΔΔCt^ to *SlEF1α* with *n* ≥ 4 per family (ANOVA with Fisher’s unprotected LSD with *p* = 0.05 on ΔCt values). **(C)** Canopy area (cm^2^) of mock-inoculated plants at 21 dpi for T2 RNAi families with (gray) and without (black) the silencing construct (*n* ≥ 3, ANOVA with Fisher’s unprotected LSD, *p* = 0.05). **(D)** Stunting of *V. dahliae-*inoculated plants when compared with the average stunting of mock-inoculated plants per genotype at 21 dpi (*n* ≥ 5, *t*-test with ^∗^*p* < 0.05). a–c: Genotypes having average values with a letter in common are not statistically significant different (at *P* = 0.05).

T2 plants derived from the three transformants, TV181034, TV181036, and TV181037, were tested for presence of the silencing construct with a NPTII- and 35S specific-PCR (Supplementary Table 1). This revealed that 7 out of 45 plants (15.5%), 17 out of 89 plants (19.1%), and 9 out of 90 plants (10.0%) of the plants of the T2 families TV181034, TV181036, and TV181037, respectively, did not carry the silencing construct (Figure 2A). When compared with plants lacking the construct (− NPTII/35S) of all families, the expression of *SlWAT1* in the roots of plants carrying the silencing construct (+ NPTII/35S) was significantly reduced, with the residual expression of on average 20.2, 54.4, and 65.5% for the family TV181036, TV181034, and TV181037, respectively (Figure 2B). We also determined whether the presence of the silencing construct affected plant growth in the absence of *V. dahliae* inoculation. No significant difference in canopy area of mock-inoculated plants was found for any of the T2 families compared with mock-inoculated MM plants (Figure 2C).

To test for loss of susceptibility to *V. dahliae*, plants from all three T2 families were challenged with *V. dahliae.* To this end, stunting based on canopy area was calculated between mock- and *V. dahliae-*inoculated plants for each of the genotypes at 21 dpi and compared with *V. dahliae*-induced stunting in MM plants (Figure 2D). Of the three tested families, a significant reduction in stunting of *V. dahliae-*inoculated plants was found only in the family TV181034 with a value of on average 32.2 vs 52.6% in *V. dahliae*-inoculated MM plants.

**FIGURE 3:**
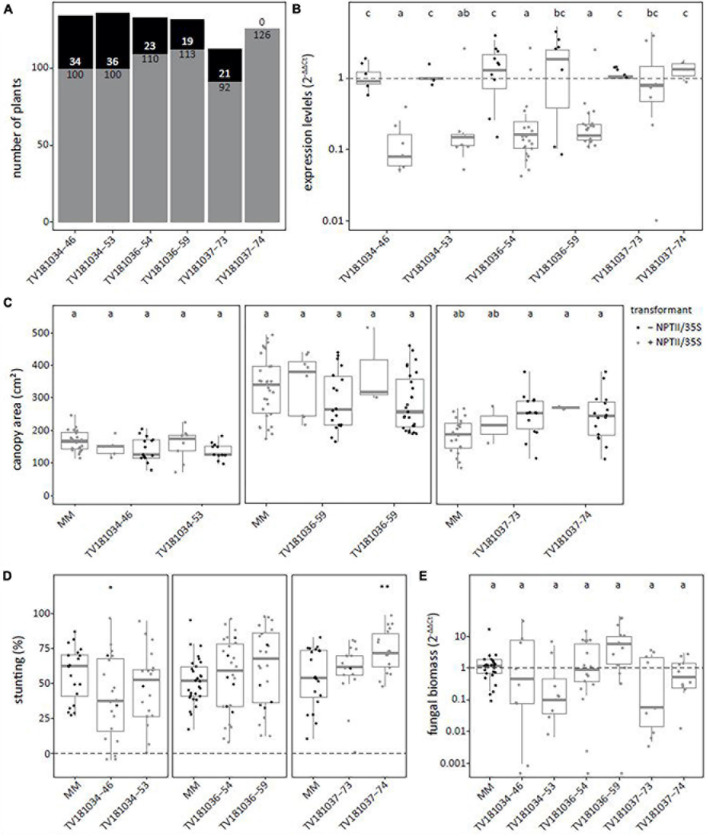
Loss of susceptibility to *V. dahliae* was not confirmed in all *SlWAT1* T3 RNAi families. **(A)** Total number of plants with (gray) and without (black) the silencing construct for six T3 RNAi families which were obtained from three different T2 RNAi families. **(B)** Expression levels of T3 *SlWAT1* RNAi families relative to plants without the silencing construct (– NPTII/35S, gray) and normalized using 2^–ΔΔCt^ to *SlEF1α* with *n* ≥ 4 per family with (gray) and without (black) the silencing construct (ANOVA with Fisher’s unprotected LDS with *p* = 0.05 on ΔCt values). **(C)** Canopy area (cm^2^) of mock-inoculated plants at 21 days post inoculation (dpi) for T3 *SlWAT1* RNAi families with (gray) and without (black) the silencing construct. Data from two independent experiments with *n* ≥ 2 (ANOVA with Fisher’s unprotected LSD, *p* = 0.05). **(D)** Stunting (%) of *V. dahliae*-inoculated plants when compared with the average stunting of mock-inoculated plants per genotype at 21 dpi. Box plots represent data with *n* ≥ 8 plants per experimental repeat (*t*-test when compared with MM with *p < 0.05 and **p < 0.01). **(E)** Relative fungal biomass in the stems of RNAi plants at 21 dpi with *V. dahliae*. This was calculated as the ratio of the *V. dahliae* ITS gene amplification in comparison with the tomato *SlRUB* gene (Supplementary Table 1) and normalized the *V. dahliae-*inoculated MM plants using 2^–ΔΔCt^ on a log10 scale with *n* ≥ 7 per family (ANOVA with Fisher’s unprotected LSD, *p* = 0.05 on ΔCt). a–c: Genotypes having average values with a letter in common are not statistically significant different (at *P* = 0.05).

To confirm the results of the T2 generation, two plants per T2 family were kept for T3 seed production. T3 plants with and without the RNAi construct were identified (Figure 3A). In the T3 families derived from T2 plants of TV181034-46 and –53 as well as TV181036-54 and –59, a significant reduction of *SlWAT1* expression to 12.0, 41.4, 35.2 and 30.6%, respectively, were found in the roots of plants carrying the RNAi constructs (Figure 3B). Unfortunately, *SlWAT1* was not silenced at all in plants carrying the RNAi construct for families TV181037-73 and –74, as *SlWAT1* expression was on average 39.4 and 36.5% higher than in control plants lacking the RNAi construct, respectively (Figure 3B). No statistically significant difference in canopy area was found for plants of any of the T3 RNAi families when compared with the canopy area of MM plants (Figure 3C). This is in agreement with the results obtained in the T2 families (Figure 2C), indicating that the presence of the silencing construct did not significantly affect plant growth at this developmental stage.

The plants of the six T3 RNAi families were challenged with *V. dahliae*, and similar levels of stunting of *V. dahliae-*inoculated plants were found for most plants of the T3 families when compared with *V. dahliae*-inoculated MM plants (Figure 2D). Only in one T3 family, TV181034-46, stunting of *V. dahliae-*inoculated plants was significantly reduced to (on average) 39.5% when compared with *V. dahliae*-inoculated MM plants that displayed an average stunting of 56.6%.

To quantify *V. dahliae* colonization in the T3 RNAi families, fungal biomass was quantified on genomic DNA by targeting the *ITS* gene of *V. dahliae* relative to the tomato reference gene *SlRUB* (Supplementary Table 1) in stems of *V. dahliae-*inoculated plants at 21 dpi for each genotype. No significant reduction in fungal biomass in plants of all six T3 RNAi families was found when compared with *V. dahliae-*inoculated MM plants (Figure 3E).

### Targeted Deletion in *SlWAT1* Leads to Loss-of-Susceptibility to *V. dahliae* Despite Severe Growth Defects

**FIGURE 4:**
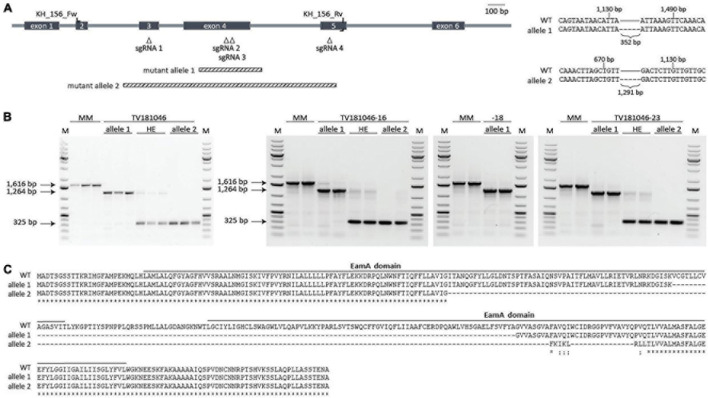
CRISPR T2 family TV181046 and its T3 progeny carry a bi-allelic mutation in *SlWAT1*. **(A)** Schematic overview of *SlWAT1* (Solyc04g080940) indicating location of the sgRNAs, primers used for genotyping and the two mutant alleles (right). Sequencing revealed a 352 and 1,291 bp deletion for mutant alleles 1 and 2, respectively, compared with *SlWAT1* wild-type (WT) (right). **(B)** Gel electrophoresis (1% TAE, ethidium bromide) of gene-specific PCR with (wild-type band at 1,616 bp) showing the two mutant alleles as well as heterozygous (HE) plants for the T2 (left) and the T3 (right) plants with a 1 kb ladder (M). **(C)** Protein alignments of SlWAT1 wild-type (WT) with mutant alleles 1 and 2 showing a 121 and 197 amino acid deletion, respectively. Mutant alleles were translated into protein using http://www.softberry.com/berry.phtml. CLUSTAL multiple sequence alignment was done using https://www.ebi.ac.uk/Tools/msa/muscle/. Solid bars indicate predicted protein domains annotated as EamA domain (https://www.ebi.ac.uk/interpro).

In order to circumvent interference of residual *SlWAT1* expression as shown for the RNAi families, we explored approaches for targeted knock-out. To this end, stable transformants using CRISPR-Cas9 were generated. The CRISPR-Cas9 construct was designed with four sgRNAs that targeted sequences in exons 3, 4, and 5 of the *SlWAT1* gene (Figure 4A). The use of multiple sgRNAs increased the possibility of creating large deletions due to the occurrence of double stranded breaks at multiple sgRNAs locations simultaneously (Do et al., 2019). T1 transformants were evaluated for the occurrence of mutations in *SlWAT1* with a gene-specific PCR and gel electrophoresis to detect aberrantly sized PCR products (Supplementary Table 1). By focusing on large deletions, small deletions, small insertions, and single nucleotide polymorphisms might have been missed. Three transformants (#10, #19, and #28) showed a relatively large deletion, while for a fourth transformant (#23) an additional band appeared above the wild-type band (Supplementary Figure 1). However, T2 seeds from only one of these mutants, TV181046 (#19), were obtained as the other transformants were either not successfully transferred from *in vitro* conditions to soil, did not set fruits, or did not produce seeds.

First, plants from the T2 CRISPR family TV181046 were genotyped to confirm the presence of a mutation by sequencing. In fact, seedlings of TV181046 were found to carry bi-allelic mutations with either a smaller deletion (allele 1), or a larger deletion (allele 2), or heterozygous plants that carry both types of deletions (Figures 4A,B). The smaller 352 bp deletion (allele 1), located in exon 4, led to a 121 amino acid deletion, while the larger 1,291 bp deletion (allele 2), spanning exons 3, 4, and partly 5, resulted in a 197 amino acid deletion. As only one T2 CRISPR line was obtained, we propagated plants with the heterozygous deletions as well as homozygous plants for each mutant allele to obtain a larger panel of genotypes (T3) for testing. Seeds from three T3 CRISPR lines were obtained, TV181046-16, –18, and –23, genotyped and found to be heterozygous for the deletions (TV181046-16 and –23) and homozygous for allele 1 (TV181046-18) (Figure 4B).

As for neither of the two deletions (allele 1 and allele 2, Figure 4C) a premature stop codon was predicted,^10^ we subsequently investigated whether these deletions affected any known domain within SlWAT1. To this end, protein domains were predicted using InterPro.^11^ For wild-type SlWAT1 two EamA domains were found. Most EamA domain-containing proteins are classified as metabolite transporters that usually carry two copies of this domain (Jack et al., 2001). For the SlWAT1 mutant alleles both predicted EamA domains were affected (Supplementary Figure 2). As *AtWAT1* is located in the tonoplast (Ranocha et al., 2010), and also because many EamA domain-containing proteins carry multiple transmembrane domains, we further predicted the transmembrane domains for wild-type SlWAT1, mutant allele 1 and mutant allele 2. Wild-type SlWAT1 was predicted to contain ten transmembrane domains which was described before for WAT1 in Arabidopsis and cotton as well (Ranocha et al., 2010; Tang et al., 2019). For mutant allele 1 only seven out of ten transmembrane domains were found and for mutant allele 2 only four (Supplementary Figure 2). Collectively, our data suggested that both mutant alleles carry a deletion which affects known domains in *SlWAT1*, presumably leading to loss of its biological function. This allows us to further study these knock-out CRISPR lines for loss of susceptibility to *V. dahliae.*

**FIGURE 5:**
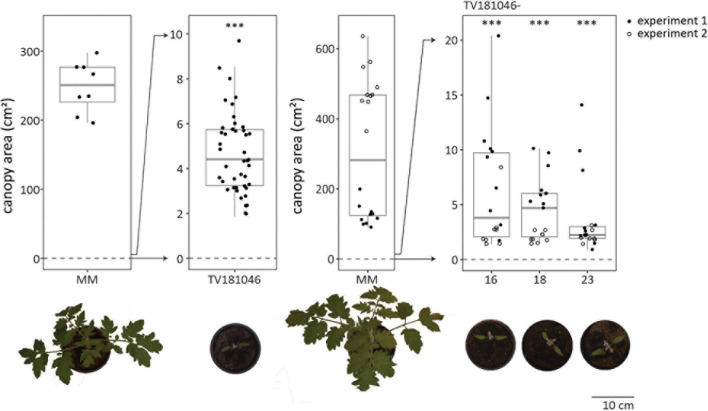
CRISPR T2 family TV181046 and its T3 progeny display severe growth and development defects. Canopy area of mock-inoculated plants at 21 dpi for T2 CRISPR line TV181046 (left) and T3 CRISPR lines TV181046-16, –18, and –23 (right). Data from one or two independent experiments with *n* ≥ 8 (*t*-test compared with MM with ^∗∗∗^*p* = 0.001).

Irrespective of the type of mutation, all plants of the T2 and the T3 generation displayed severe growth and development defects (in absence of *V. dahliae* inoculation); the germination rate was low, seedlings were small and light in color, and overall plant growth remained severely compromised (Figure 5 and Supplementary Figure 3). To quantify the size difference, we determined canopy area of mock-inoculated plants for all genotypes in the absence of *V. dahliae* inoculation. For MM Plants, canopy area of mock-inoculated plants was heavily reduced when compared with mock-inoculated plants measured at 21 dpi. While the canopy area of mock-inoculated MM plants was 300 cm^2^ on average, the canopy area of most mock-inoculated CRISPR T2 and 3 plants was less than 10 cm^2^. However, the observed aberrations alleviated slowly during further plant development and even though the CRISPR plants remained smaller than MM plants, they developed flowers and set fruits (Supplementary Figure 3).

**FIGURE 6:**
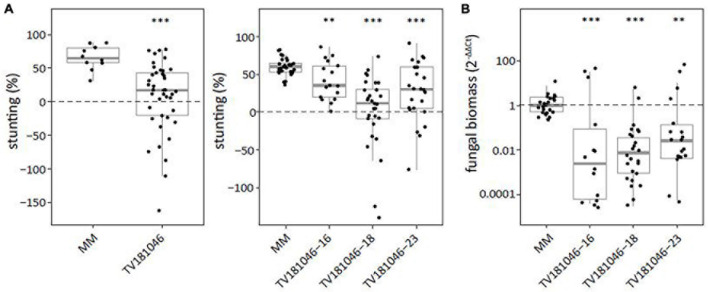
Targeted knockout of *SlWAT1* lead to loss of susceptibility to *V. dahliae.* **(A)** Stunting (%) of *V. dahliae* inoculated T2 (left) and T3 (right) plants when compared with the average stunting of mock-inoculated plants at 21 days post inoculation (dpi). Box plots represent data with *n* ≥ 9 plants per experimental repeat (*t*-test when compared with MM with ^∗∗^*p* < 0.01 and ^∗∗∗^*p* < 0.001). **(B)** Fungal biomass of *V. dahliae-*inoculated T3 CRISPR plants of all three lines in stems at 21dpi. This was calculated as the ratio of the *V. dahliae* ITS gene amplification in comparison with the tomato reference *SlRUB* gene (Supplementary Table 1) and normalized to *V. dahliae-*inoculated MM plants using 2^–ΔΔCt^ on a log10 scale with *n* ≥ 9 repeat per experimental repeat (*t*-test on ΔCt when compared with MM with ^∗∗^*p* < 0.01 and ^∗∗∗^*p* < 0.001).

To test for loss of susceptibility, plants of the T2 and the T3 generation were inoculated with *V. dahliae*. Stunting based on canopy area was calculated between mock- and *V. dahliae-*inoculated plants for each of the genotypes. Stunting of *V. dahliae*-inoculated T2 plants was significantly reduced to on average 7.1% for line TV181046 when compared with *V. dahliae-*inoculated MM plants with on average 65.5% stunting (Figure 6A and Supplementary Figure 4). In the T3 families, stunting of TV181046- 16–, –18, and –23 was significantly reduced to 41.7, 1.4, and 26.2% on average, respectively, compared with *V. dahliae-*induced stunting of 60.0% on average in MM plants. Due to the stunting calculations being based on the average of mock-inoculated plants and due to variation in plant size observed in the mutant lines, the differences in stunting of *V. dahliae*-inoculated plants were pronounced in the mutant lines when compared with the MM plants. To quantify the effect on *V. dahliae* proliferation, fungal biomass was determined in stems of *V. dahliae-*inoculated plants at 21 dpi for each genotype. In the *V. dahliae-*inoculated plants of all CRISPR T3 families, fungal biomass was significantly reduced to around 1% of the biomass in the *V. dahliae-*inoculated MM plants (Figure 6B).

### Targeted Deletion in *SlWAT1* Leads to Loss-of-Susceptibility to *V. albo-atrum* and *Fusarium oxysporum* f. sp. *Lycopersici*

**FIGURE 7:**
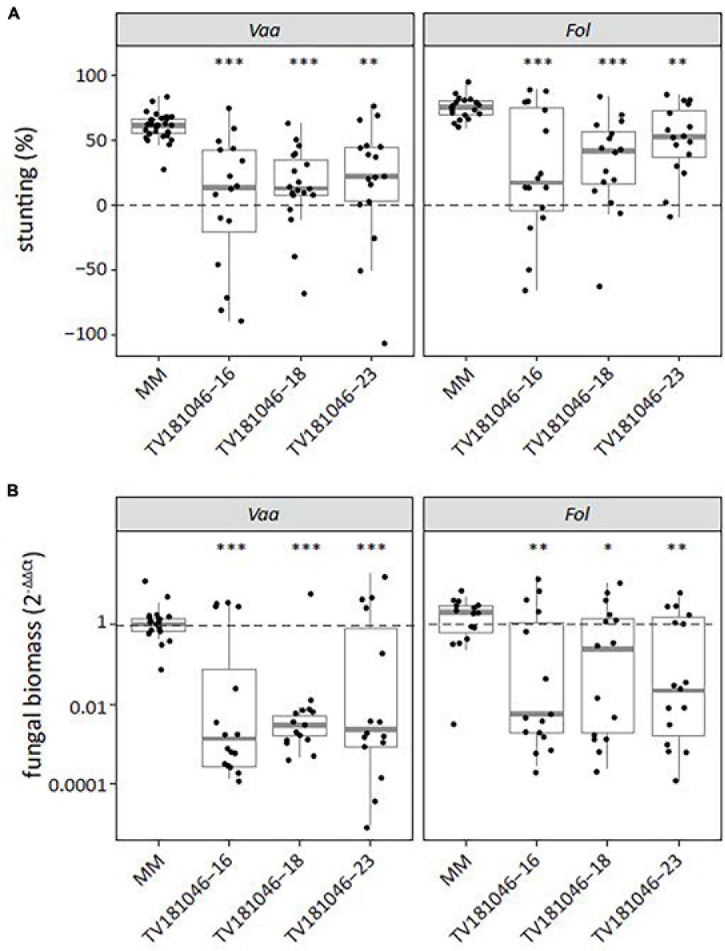
Knockout of *SlWAT1* lead to loss-of-susceptibility to *V. albo-atrum* and *Fusarium oxysporum* f. sp. *lycopersici.* **(A)** Stunting (%) of *V. albo-atrum* (*Vaa*) and *F. oxysporum* f. sp. *lycopersici* (*Fol*) inoculated T3 plants when compared with the average stunting of mock-inoculated plants at 21 days post inoculation (dpi). Box plots represent data with *n* ≥ 7 plants per experimental repeat (*t*-test when compared with MM with ^∗∗^*p* < 0.01 and ^∗∗∗^*p* < 0.001). **(B)** Fungal biomass of *Vaa-* and *Fol-*inoculated T3 CRISPR plants at 21 dpi. This was determined on genomic DNA targeting the *ITS* gene (*Vaa* and *Fol*) relative to the tomato reference gene *SlRUB* (Supplementary Table 1) and then normalized to *Vaa* and *Fol* inoculated MM plants using 2^–ΔΔCt^ on a log10 scale with *n* ≥ 7 per experimental repeat (*t*-test on ΔCt when compared with MM with ^∗^*p* < 0.05, ^∗∗^*p* < 0.01, and ^∗∗∗^*p* < 0.001).

As *S* gene-mediated resistance can lead to broad-spectrum resistance to multiple pathogens (Wang et al., 2018), we also challenged T3 CRISPR plants with *V. albo-atrum* and *Fol.* For both pathogens, inoculated T3 CRISPR plants showed significantly reduced stunting when compared with inoculated MM plants (Figure 7A). Moreover, fungal biomass was significantly reduced in *V. albo-atrum-* and *Fol*-inoculated T3 CRISPR plants of all three lines when compared with inoculated MM plants (Figure 7B).

## Discussion

For vascular pathogens such as *V. dahliae*, for which only few sources of monogenic resistance are known, crop protection mainly relies on alternative strategies. The impairment of *S* genes has gained increasing attention in resistance breeding over the last years (Pavan et al., 2010; Gawehns et al., 2013; van Schie and Takken, 2014), particularly in the light of recent advances in genome editing in plants (Andolfo et al., 2016; Langner et al., 2018; Zaidi et al., 2018; Yin and Qiu, 2019). Here, we show that targeted deletion of *SlWAT1* using CRISPR-Cas9 led to loss of susceptibility to *V. dahliae* in tomato. Plants of T3 CRISPR lines showed reduced disease symptoms upon challenge with *V. dahliae* as well as reduced fungal biomass when compared with susceptible MM plants (Figure 6). The loss of susceptibility to *V. dahliae*, as observed in plants of the CRISPR lines, could not be demonstrated consistently in plants carrying the RNAi silencing construct (Figures 1, 2). This can likely be attributed to the relatively high degree of residual *WAT1* expression in most plants of the T2 and T3 RNAi lines, which likely compromised the efficacy of silencing too much to monitor effects on *V. dahliae* infection.

In *WAT* studies in Arabidopsis and cotton, reduced Verticillium wilt symptoms and reduced fungal proliferation were observed in knock-out mutants or upon transient silencing of *WAT1*, respectively (Denancé et al., 2013; Tang et al., 2019). Remarkably, the loss of susceptibility in Arabidopsis *wat1* mutants was further extended to other vascular pathogens including bacteria and fungi (Denancé et al., 2013). *S* gene-mediated broad-spectrum resistance to multiple pathogens was described before (Wang et al., 2018), and highlights the potential of using impaired *S* genes for the control of multiple pathogens simultaneously. In fact, we also demonstrated loss of susceptibility of knock-out *slwat1* mutants to another pathogenic *Verticillium* species, *V. albo-atrum*, as well to another vascular pathogen, *Fol* (Figure 7). Also for these pathogens, disease symptoms and fungal biomass were significantly reduced when compared with susceptible MM plants. Together, this indicates that the function of *WAT1* in susceptibility to different vascular pathogens seems to be conserved across plant species, and therefore impairment of *WAT1* might offers an approach to combat different vascular pathogens in multiple crops.

To date, the function of *WAT1* in so called “vascular immunity” remains to be elucidated. *WAT1* was originally identified in a cell wall mutant screening in zinnia (Z*innia elegans*) (Pesquet et al., 2005; Ranocha et al., 2010) and the homolog of Arabidopsis was shown to be a tonoplast-localized auxin transporter (Ranocha et al., 2010). In Arabidopsis *wat1* mutants, cell wall-related phenotypes in stems were described with altered cell elongation and reduced secondary cell walls of fiber cells, hence its name *Walls Are Thin 1*. Furthermore, *wat1* mutants showed altered contents of auxin (indole-3-acetic acid, IAA), tryptophan and salicylic acid (SA) (Ranocha et al., 2010; Denancé et al., 2013). The IAA content in roots was reduced in *wat1* mutants while the SA content was found to be elevated when compared with wild-type plants (Ranocha et al., 2010), which is in line with the previously described antagonism of auxin and SA in plant immunity and development (Wang et al., 2007; Robert-Seilaniantz et al., 2011). SA does not seem to play a role in basal plant defense against *V. dahliae* as different Arabidopsis mutants with a deficiency in SA signaling, such as *enhanced disease susceptibility* (*eds1-2* and *eds5-1*), *nonexpresser of PR genes* (*npr1-1* and *npr1-3*), and *phytoalexin deficient 4* (*pad4-1*), show similar symptoms and levels of fungal biomass as control plants upon *V. dahliae* infection (Pantelides et al., 2010; Fradin et al., 2011). In contrast, a role was assigned to auxin in *V. dahliae* susceptibility as two auxin receptor mutants, *auxin signaling F-box 1* and *3* (*afb1* and *afb3*), as well as auxin transporter mutant *auxin resistant 4* (*axr4*) display reduced symptoms and less fungal biomass upon challenge with *V. dahliae* (Fousia et al., 2018). For another vascular wilt pathogen, *F. oxysporum*, two transcription factor mutants, *auxin response factor 1* and *2* (*arf1* and *arf2*), showed significantly less disease levels although fungal biomass was not quantified (Lyons et al., 2015). Collectively, auxin seems to play a crucial role in *V. dahliae* susceptibility to vascular wilt fungi, and therefore auxin-related genes may be further studied to test their potential as susceptibility factors for *V. dahliae.*

Even though *SlWAT1* CRISPR plants of tomato showed loss of susceptibility to *V. dahliae*, *V. albo-atrum*, and *F. oxysporum*, the targeted deletion in *SlWAT1* was accompanied by severe growth defects. It may be argued that the significantly reduced stunting and reduced fungal biomass accumulation in the *SlWAT1* CRISPR plants is an indirect consequence of the dramatically impaired growth and development of these mutants. In our experiments, we have performed fungal inoculations on 10-day-old seedlings. Since the observed developmental aberrations alleviated slowly during further plant development, it may be worthwhile to inoculate tomato *SlWAT1* CRISPR plants at a later time point than the control plants, when both plants have a similar overall appearance, for example by inoculating 1-month-old *SlWAT1* CRISPR plants. However, this approach obviously has the downside that mutant and control plants will not be tested at the same age.

Impairment of *S* genes is known to cause pleiotropic side effects in some cases (Clough et al., 2000; Sun et al., 2016), and also for *WAT1* such effects were described in other plant species. For Arabidopsis *wat1* mutants, no abnormalities were found in early stages of development, but older plants were stunted when compared with wild-type plants (Ranocha et al., 2010). Transient silencing of *WAT1* in cotton resulted in reduced root length and shorter first internodes (Tang et al., 2019). Such growth defects can certainly be attributed to the imbalance between auxin and SA. Firstly, it is well known that auxin plays an essential role in many aspects of plant development (Korver et al., 2018) and its downregulation, as shown in Arabidopsis *wat1* mutants, might negatively affect growth. Secondly, Arabidopsis *wat1* mutants also showed higher SA levels, which is known to affect plant growth as observed in the *constitutive expressor of PR genes 5* (*cpr5*) mutant which shows high SA levels accompanied by severe dwarfism (Bowling et al., 1997). Evidently, pleiotropic effects of impaired *S* genes are not desirable for breeding purposes, as it might affect yield but also overall development (Hückelhoven et al., 2013; Engelhardt et al., 2018). Additionally, special attention should also be given to resistance against other pathogens as an altered hormone balance, as observed in *WAT1-*mediated resistance (Denancé et al., 2013), can influence resistance to other pathogens (Thomma et al., 1998). Therefore, alternatives for obtaining mutants without such pleiotropic effects need to be explored. For example, potential natural allelic variants of *WAT1* in wild germplasm that can no longer be exploited by the pathogen, but that do not display pleiotropic effects, could be used for breeding. Alternatively, mutant populations can be used to identify *wat1* mutants omitting the severe growth defects. Certainly, mutants with smaller targeted deletions or even single base pair changes could also be studied, to find essential domains that are only required by *Verticillium* spp. for disease development, but that are not involved in tomato growth and development. Lastly, targeted modification in the promoter region of a *S* gene could circumvent pleiotropy by preventing binding the pathogen effector, as it was demonstrated by the *xa13-*mediated resistance against bacterial blight (Chu et al., 2006; Zaka et al., 2018).

In the case of *WAT1*, two Arabidopsis *wat1* mutant alleles that each carry a T-DNA insertion have been described (Ranocha et al., 2010). The T-DNA insertion located 55 bp upstream of the ATG translation start codon leads to lack of *WAT1* expression in the *wat1-1* mutant line. In contrast, the *wat1-2* mutant line that carries the T-DNA insertion 28 bp downstream of the stop codon has about 50% residual *WAT1* expression when compared with wild-type plants. Compared with *wat1-1*, a less strong phenotype was displayed by *wat1-2* plants (Ranocha et al., 2010). In our study, the tomato *WAT1* RNAi plants had a similar canopy area to the control MM plants until 6 weeks after sowing (Figure 2C). This finding may indicate that the residual *WAT1* expression in the tomato RNAi lines is sufficient to prevent negative effects on plant growth. Unfortunately, the *WAT1* expression levels were not sufficiently reduced to hamper *Verticillium* infection in most RNAi tomato plants, since a significant reduction in stunting upon *V. dahliae* inoculation was only observed in the T2 family TV181034 (Figure 2) and the derived T3 family TV181034-46 (Figure 3). Variation in stunting was shown between individual T2 and T3 plants, with some plants showing a very low level of stunting (Figures 2, 3), which may be associated with variation in residual *WAT1* expression. Therefore, in further studies, it could be worthwhile to determine the residual level of *WAT1* expression and correlate that with stunting levels of individual RNAi plants in order to assess a direct correlation between these characteristics. Such direct correlation justifies the further search for a natural *wat1* alleles or the generation of mutations in the promoter of the *WAT1* gene that can associate reduced *WAT1* expression with reduced *Verticillium*-induced stunting.

Summarizing, it remains challenging to identify *WAT1* alleles in tomato, as well as in other crops, that cannot be exploited by *Verticillium* spp. for disease development, yet that do not negatively impact plant growth and development.

## Data Availability Statement

The original contributions presented in the study are included in the article/Supplementary Material, further inquiries can be directed to the corresponding author/s.

## Author Contributions

KH, HS, BT, and YB conceived the study and wrote the manuscript. KH designed and performed the experiments and analyzed the data. DS, SC, and MO helped in collecting data. All authors read and approved the manuscript.

## Conflict of Interest

The authors declare that the research was conducted in the absence of any commercial or financial relationships that could be construed as a potential conflict of interest.

## Publisher’s Note

All claims expressed in this article are solely those of the authors and do not necessarily represent those of their affiliated organizations, or those of the publisher, the editors and the reviewers. Any product that may be evaluated in this article, or claim that may be made by its manufacturer, is not guaranteed or endorsed by the publisher.

## Footnotes

1
https://crispr.cos.uni-heidelberg.de/

2
http://www.endmemo.com/bio/gc.php

3
http://unafold.rna.albany.edu/?q=mfold/RNA-Folding-Form

4
https://sgrnascorer.cancer.gov/

5
https://portals.broadinstitute.org/gpp/public/analysis-tools/sgrna-design

6
http://crispr.wustl.edu/

7
https://www.addgene.org/

8
https://www.arabidopsis.org/

9
https://solgenomics.net/tools/blast/

10
http://www.softberry.com/berry.phtml

11
https://www.ebi.ac.uk/interpro/

## Supplementary Material

Supplementary Figure 1 Gel electrophoresis (1% TBE, Gelred, image colors inverted) of gene-specific PCR with primers KH_156 on primary transformants (T1) of plants transformed with *SlWAT1* CRISPR-Cas9 construct. Wild-type PCR product (1,616 bp) indicated with an arrow and highlighted are mutants #10, #19, #23, and #28 that were transferred to the greenhouse, and of which seeds were only obtained from #19 (TV181046).

Supplementary Figure 2 Predicted transmembrane domains for wild-type SlWAT1 (left), mutant allele 1 (right), and mutant allele 2 (bottom). Graphs were generated with TMHMM Server v. 2.0 (http://www.cbs.dtu.dk/services/TMHMM/).

Supplementary Figure 3 CRISPR T2 family TV181046 and its T3 progeny TV181046-16, –18, and –23 display severe growth and development defects. Pictures of MM and T3 CRISPR WAT1 plants at different time points.

Supplementary Figure 4 Canopy area pictures mock- and *V. dahliae-*inoculated (strain JR2) plants for CRISPR T2 family TV181046 and T3 families TV181046-16, –18, and –23 at 21 dpi compared to MM plants of respective experiment.

Supplementary Table 1 Primers used in this study.

Supplementary Table 2 Overview of primary transformants (T1) with presence/absence of silencing construct (NPTII/35S), relative *SlWAT1* expression normalized to control plants at 1 (2^–ΔΔCt^) and plants from which T2 seeds were obtained.
