# Supplementary figures and images for: Impairment of Tomato WAT1 Enhances Resistance to Vascular Wilt Fungi Despite Severe Growth Defects
Source: Front Plant Sci. 2021 Sep 13;12:721674. doi: 10.3389/fpls.2021.721674 (PMC8473820; doi:10.3389/fpls.2021.721674)

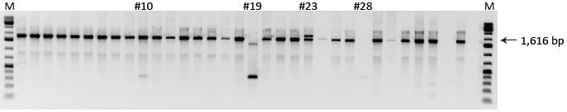

Supplement: Supplementary Figure 1 — Gel electrophoresis (1% TBE, Gelred, image colors inverted) of gene-specific PCR with primers KH_156 on primary transformants (T1) of plants transformed with SlWAT1 CRISPR-Cas9 construct. Wild-type PCR product (1,616 bp) indicated with an arrow and highlighted are mutants #10, #19, #23, and #28 that were transferred to the greenhouse, and of which seeds were only obtained from #19 (TV181046). [file Image_1.JPEG]

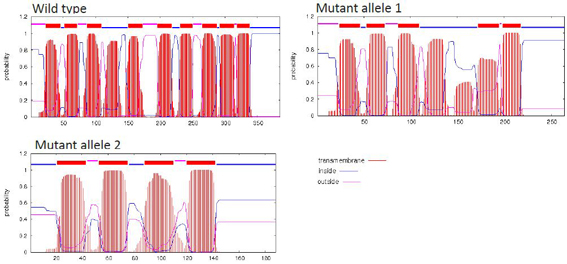

Supplement: Supplementary Figure 2 — Predicted transmembrane domains for wild-type SlWAT1 (left), mutant allele 1 (right), and mutant allele 2 (bottom). Graphs were generated with TMHMM Server v. 2.0 (http://www.cbs.dtu.dk/services/TMHMM/). [file Image_2.JPEG]

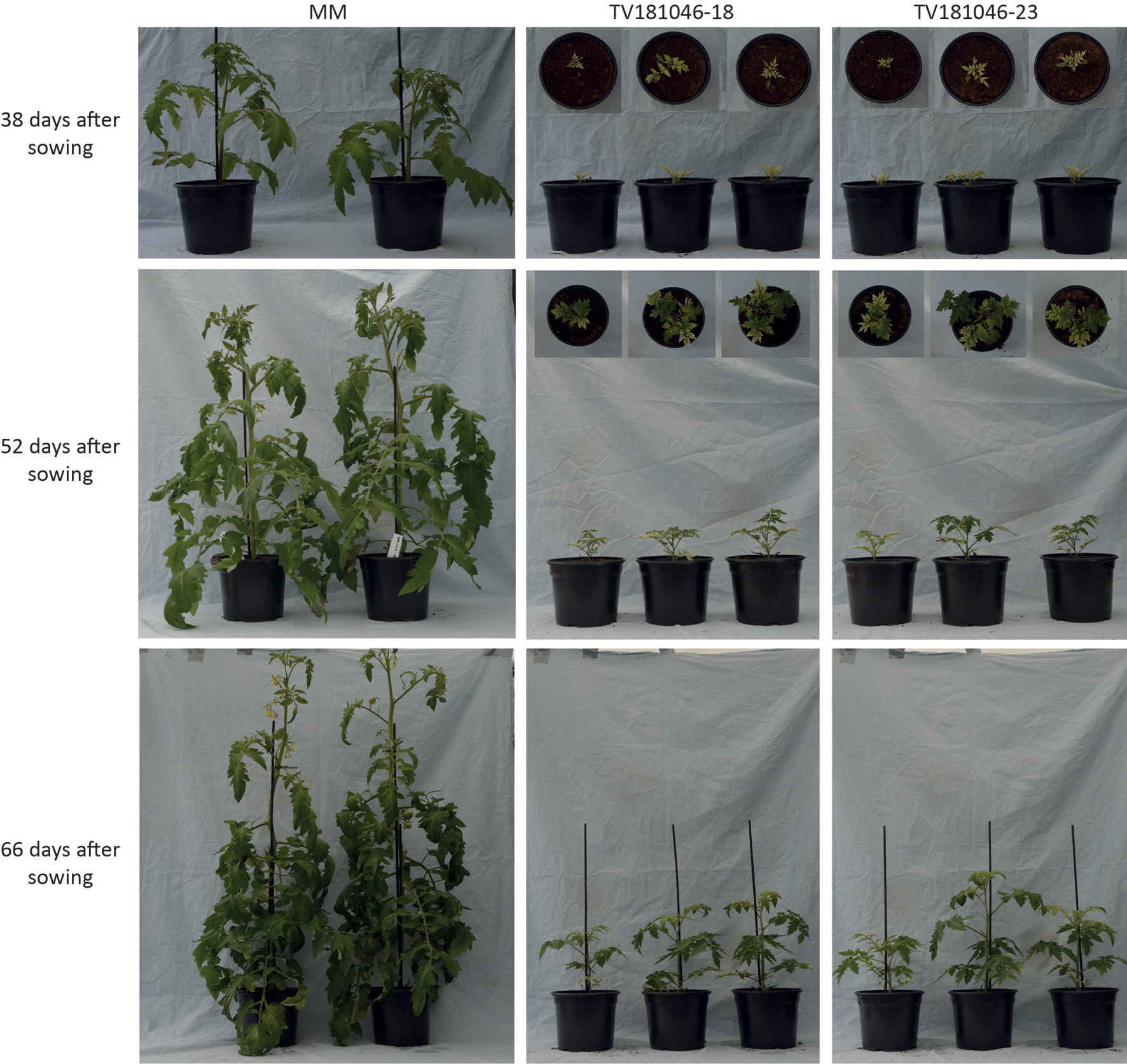

Supplement: Supplementary Figure 3 — CRISPR T2 family TV181046 and its T3 progeny TV181046-16, –18, and –23 display severe growth and development defects. Pictures of MM and T3 CRISPR WAT1 plants at different time points. [file Image_3.JPEG]

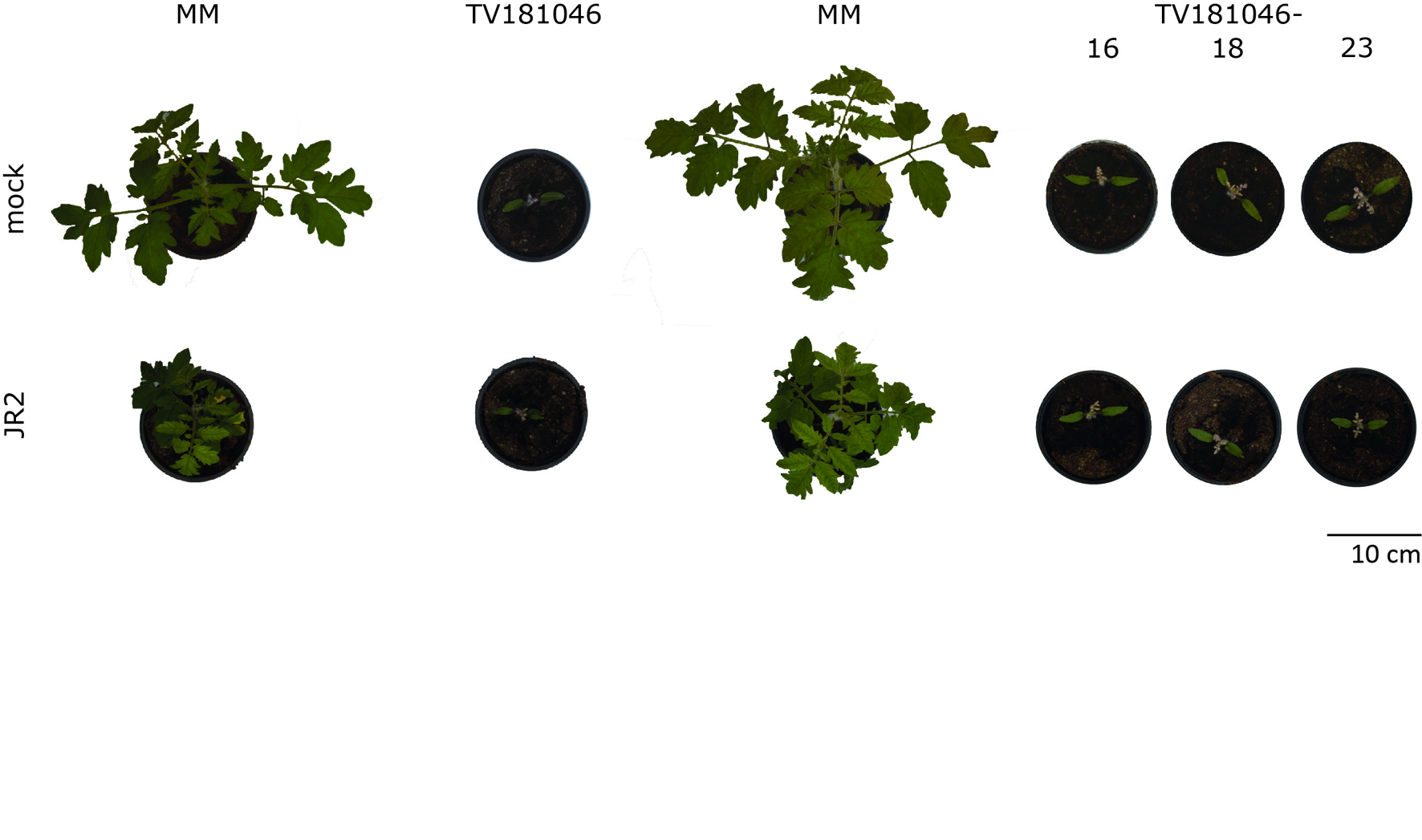

Supplement: Supplementary Figure 4 — Canopy area pictures mock- and V. dahliae-inoculated (strain JR2) plants for CRISPR T2 family TV181046 and T3 families TV181046-16, –18, and –23 at 21 dpi compared to MM plants of respective experiment. [file Image_4.JPEG]
