# Supplementary material for: Impairment of Tomato WAT1 Enhances Resistance to Vascular Wilt Fungi Despite Severe Growth Defects
Source: Front Plant Sci. 2021 Sep 13;12:721674. doi: 10.3389/fpls.2021.721674 (PMC8473820; doi:10.3389/fpls.2021.721674)
Supplement: Supplementary Table 1 — Primers used in this study. [file Table_1.DOCX]

**Supplementary Table S1 |** Primers used in this study.

| **Primer** | **Sequence (5’→ 3’)*** | **Used for** |
| --- | --- | --- |
| SlEF1α_Fw | ATTGGAAACGGATATGCCCCT | RT-qPCR (reference tomato) |
| SlEF1α_Rv | TCCTTACCTGAACGCCTGTCA |  |
| WAT1_qPCR_Fw  WAT1_qPCR_Rv | GGGGGTCCAGTTTTTGTTGC  CTCCGATTATCCCGCCCAAG | RT-qPCR (*SlWAT1* expression) |
| KH_093_Fw  KH_093_Rv | caccCGGCCCAACAATTTACAGCCC  GAACTAGCCAAGCCTGAGGG | RNAi construct |
| MA_NPTII_421_Fw  MA_NPTII_421_Rv | GAAGGGACTGGCTGCTATT  AATATCACGGGTAGCCAAC | RNAi construct NPTIS |
| MA_35S_597_Fw  MA_35S_597_Rv | TACAAAGGCGGCAACAAAC  AGCAAGCCTTGAATCGTCC | RNAi construct 35S |
| SlWat1_3.02_Fw  SlWat1_4.06_Fw  SlWat1_4.23_Fw  SlWat1_5.04_Fw | GTATGGCAGAAGCAAAAGTA  CTAGGCTCTCGGTCACGTCG  CGGGTACTTCTTGAGTACGG  ATATGGTGCATTGACAGAGG | sgRNAs CRISPR |
| KH_156_Fw  KH_156_Rv | CAGGAAAGACAGGCCACAACT  CCTAACGCGAAGGAAGCCAT | genotyping |
| SlRub_QPCR_F  SlRub_QPCR_R | GAACAGTTTCTCACTGTTGAC  CGTGAGAACCATAAGTCACC | Tomato rubisco gene |
| Vd-ITS-Fw  Vd-ITS-Rv | AAAGTTTTAATGGTTCGCTAAGA  CTTGGTCATTTAGAGGAAGTAA | *V. dahliae* biomass |
| FolTub_F: | CTCTGGCAACAAGTATGTTCCC | F. oxysporum Tubulin (biomass) |
| FolTub_R: | TTGTCGGGACGGAAGAGCTGA |  |
