# Supplementary material for: Impairment of Tomato WAT1 Enhances Resistance to Vascular Wilt Fungi Despite Severe Growth Defects
Source: Front Plant Sci. 2021 Sep 13;12:721674. doi: 10.3389/fpls.2021.721674 (PMC8473820; doi:10.3389/fpls.2021.721674)
Supplement: Supplementary Table 2 — Overview of primary transformants (T1) with presence/absence of silencing construct (NPTII/35S), relative SlWAT1 expression normalized to control plants at 1 (2–ΔΔCt) and plants from which T2 seeds were obtained. [file Table_2.docx]

**Supplementary Table S2 |** Overview of primary transformants (T1) with presence/absence of silencing construct (NPTII/35S), relative *SlWAT1* expression normalized to control plants at 1 (2^-ΔΔCt^) and plants from which T2 seeds were obtained.

| **Plant** | **NPTII/35S** | **2^-ΔΔCt^** | **Seed production** |
| --- | --- | --- | --- |
| #93-1 | - | 0.61 | NA |
| #93-2 | + | 0.11 | Yes (no fruits) |
| #93-3 | - | 0.54 | NA |
| #93-4 | - | 0.98 | NA |
| #93-5 | + | 1.35 | NA |
| #93-6 | + | 0.59 | NA |
| #93-7 | - | 2.70 | NA |
| #93-8 | + | 1.65 | NA |
| #93-9 | + | 2.04 | NA |
| #93-10 | - | 0.87 | NA |
| #93-11 | + | 0.47 | Yes (TV181034) |
| #93-12 | - | 0.62 | NA |
| #93-13 | + | 0.29 | Yes (no fruits) |
| #93-14 | + | 0.46 | Yes (TV181036) |
| #93-15 | - | 1.01 | NA |
| #93-16 | NA | 0.23 | Yes (TV181037) |
